# Supplementary material for: Human Sperm Head Vacuoles Are Related to Nuclear-Envelope Invaginations
Source: Int J Mol Sci. 2023 Jun 12;24(12):10027. doi: 10.3390/ijms241210027 (PMC10298367; doi:10.3390/ijms241210027)
Supplement: Supplementary file 1 [file ijms-24-10027-s001.zip › ijms-2401350-supplementary.pdf]

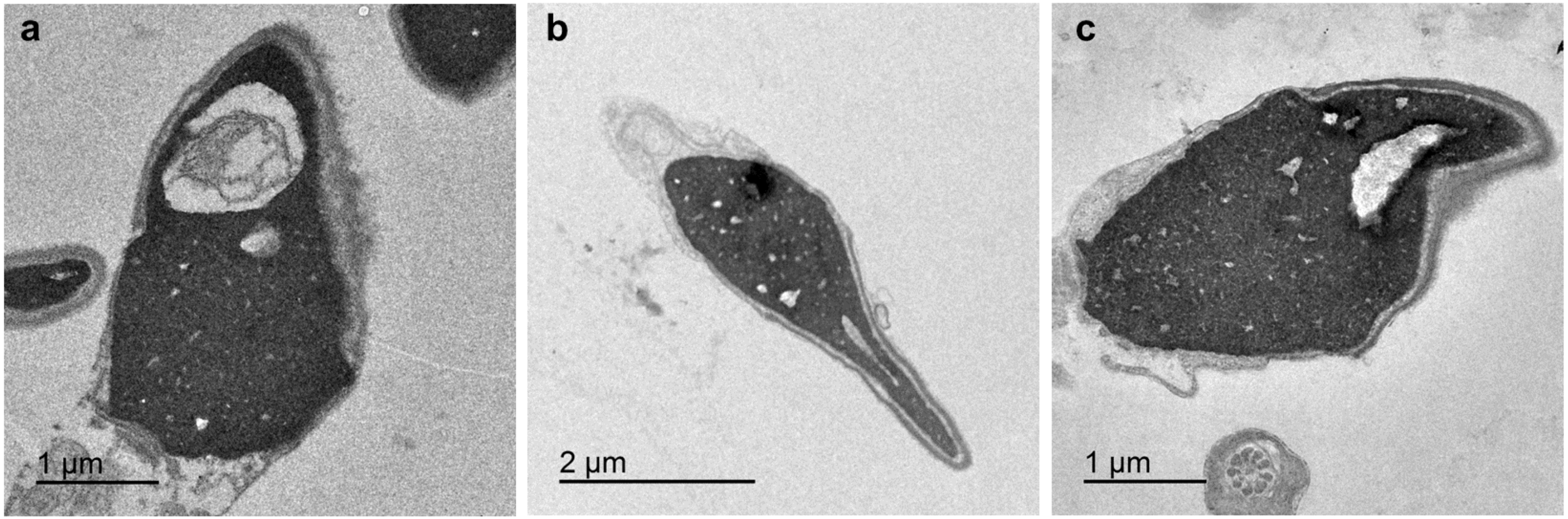


**Figure S1.** Transmission electron microscopy (TEM) micrographs of longitudinal sections of human spermatozoa showing negative controls. **(a)** Negative control omitting polyclonal anti-actin antibody. **(b)** Negative control omitting polyclonal anti-calicin antibody. **(c)** Negative control omitting monoclonal anti-CuZn-SOD antibody. Immunostainings were absent.
